# Self-Consistent Charge Density Functional Tight-Binding Study of Poly(3,4-ethylenedioxythiophene): Poly(styrenesulfonate) Ammonia Gas Sensor

**DOI:** 10.1186/s11671-017-1878-2

**Published:** 2017-02-06

**Authors:** Ampaiwan Marutaphan, Yotsarayuth Seekaew, Chatchawal Wongchoosuk

**Affiliations:** 1 Department of Physics, Faculty of Science, Kasetsart University, 10900 Chatuchak, Bangkok Thailand; 2 Faculty of Science and Technology, Rajamangala University of Technology Suvarnabhumi, 11000 Nonthaburi, Thailand

**Keywords:** PEDOT:PSS, Conducting polymers, Ammonia gas sensor, SCC-DFTB, QM/MD simulation

## Abstract

Geometric and electronic properties of 3,4-ethylenedioxythiophene (EDOT), styrene sulfonate (SS), and EDOT: SS oligomers up to 10 repeating units were studied by the self-consistent charge density functional tight-binding (SCC-DFTB) method. An application of PEDOT:PSS for ammonia (NH_3_) detection was highlighted and investigated both experimentally and theoretically. The results showed an important role of H-bonds in EDOT:SS oligomers complex conformation. Electrical conductivity of EDOT increased with increasing oligomers and doping SS due to enhancement of *π* conjugation. Printed PEDOT:PSS gas sensor exhibited relatively high response and selectivity to NH_3_. The SCC-DFTB calculation suggested domination of direct charge transfer process in changing of PEDOT:PSS conductivity upon NH_3_ exposure at room temperature. The NH_3_ molecules preferred to bind with PEDOT:PSS via physisorption. The most favorable adsorption site for PEDOT:PSS-NH_3_ interaction was found to be at the nitrogen atom of NH_3_ and hydrogen atoms of SS with an average optimal binding distance of 2.00 Å.

**Electronic supplementary material:**

The online version of this article (doi:10.1186/s11671-017-1878-2) contains supplementary material, which is available to authorized users.

## Background

Poly(3,4-ethylenedioxythiophene) (PEDOT) is one of the most promising π-conjugated polymers. Because of its unique properties such as low redox potential [1], low band gap (1.5–1.6 eV) [2], and good stability (below 150 °C) [3], PEDOT can be used in several applications such as transparent electrodes [4, 5], printing circuit boards [6, 7], OLED displays [8, 9], solar cell [10, 11], and textile fibers [12]. To improve the solubility and conductivity of PEDOT, poly(styrenesulfonate) (PSS) as a dispersant and a charge-balancing dopant is usually doped into PEDOT during the polymerization [10, 13–16]. Combination of PEDOT and PSS (PEDOT:PSS) provides the enhanced electrical conductivity (1–10 S•cm^−1^) with solubility in water which allows the conductive polymer to be easily-processed as an electronic ink for practical applications in field of printed electronics [17].

In theoretical studies, structural and electronic properties of PEDOT and PEDOT:PSS have been investigated by many research groups, i.e., Dkhissi et al. used ab initio Hartree–Fock (HF/6-31G) and density functional theory (DFT/6-31G) methods to exhibit relative stability of the aromatic and quinoid forms of neutral PEDOT in the ground state [18, 19]. Aleman et al. reported structural and electronic properties of n-EDOT with *n =* 1–8 [20]. Lenz et al. studied the influence of the degree of doping on the reflectivity and optical properties of PEDOT:PSS based on GGA PW91 functional [14]. Very recently, Gangopadhyay investigated the nature of the interaction between PEDOT and PSS using B3LYP/6-31G** [21]. However, to our best knowledge, there has been no report on theoretical studies of PEDOT:PSS for ammonia sensing applications.

Ammonia (NH_3_) is highly toxic gas that is naturally existed in the atmosphere at low-ppb to sub-ppb levels. It can be widely used in various applications such as production of fertilizer and chemicals, refrigeration systems, and clinical diagnosis [22]. However, at high concentration of NH_3_, it can cause irritation the skin, eyes, nose, throat to respiratory tract due to its corrosive properties. Exposure to a massive concentration of NH_3_ (>5000 ppm) may be fatal within minutes. Therefore, detection of NH_3_ has attracted much attention for environment protection and human health. Recently, several research groups have reported the fabrication of NH_3_ gas sensors based on inorganic, organic and hybrid materials. For example, Pang et al. synthesized cellulose/TiO_2_/PANI composite nanofibers by electrospinning and polymerization for NH_3_ detection at room temperature [23]. The response value of the composite nanofibers to 250 ppm NH_3_ was found to be 6.335. Zhang et al. fabricated MoS_2_/ZnO nanocomposite film sensor by layer-by-layer self-assembly technique. The MoS_2_/ZnO nanocomposite film exhibited a high sensitivity to NH_3_ with a normalized response value of 24.38% in gas concentration of 5 ppm at room temperature [24]. Moon et al. prepared Co_3_O_4_–SWCNT nanocomposites by arc-discharge method [25]. The Co_3_O_4_-SWCNT sensor was investigated to various reducing gases such as H_2_S, NH_3_, H_2_, and CH_4_. At the optimum operating temperature of 250 °C, the response value of Co_3_O_4_-SWCNT sensor was ~50% for 100 ppm NH_3_ detection. Other current materials for NH_3_ sensing application were summarized in Table 1. Although some materials with specific preparation methods exhibited excellent sensing performances towards NH_3_, most of them did not support the preparation of sensing film on flexible substrate that is one of serious problems for future wearable gas sensing application. In addition, each of these methods suffers from several disadvantages such as high cost, high complexity, long operating time for sensing film preparation and high operating temperature in gas detection. Therefore, the development of NH_3_ gas sensors on flexible substrate with high sensitivity, simplicity, low temperature processing, high productivity, low-cost, low material waste and room operating temperature for NH_3_ detection is still an important task for low-cost high-performance wearable gas sensors. In this work, we have fabricated a PEDOT:PSS NH_3_ gas sensor based on inkjet printing method. Theoretical studies of PEDOT:PSS for NH_3_ detection have been performed for the first time by using Self-consistent charge density functional tight-binding (SCC-DFTB). The most favorite site of NH_3_ adsorption on PEDOT:PSS have been systematically investigated. It should be noted that the SCC-DFTB method was derived from DFT by neglect, approximation, and parametrization of interaction integrals. It offers several advantages including rapid computation of large scale molecular systems (several thousands of atoms), reliable description of dispersions and weak interactions (Van der Waals and H-bonding), and good prediction for properties (geometry, electronics, and binding energies) [26–28]. Moreover, the SCC-DFTB method was used for investigation of NH_3_ adsorption on sensing material, which is consistent with experimental observations [29]. The SCC-DFTB was therefore selected for PEDOT:PSS theoretical studies on NH_3_ sensing application for this work.

**Table 1:** Comparison of sensing materials for NH_3_ detection in the literatures with the present work

Sensing material	Gas response	NH_3_(ppm)	Operating temperature	Ref.
Reduce graphene oxide	0.64% (ΔR/R_0_)	1000	22 °C	[50]
Silver Nanocrystal-MWCNTs	~9% (ΔR/R_0_)	10,000 (1%)	RT.	[51]
PANI	2.3% (Δρ/ρ_air_)	750	RT.	[52]
ZnO nanorods	10.1 (R_a_/R_g_)	100	~300 °C	[53]
SnO_2_	1.74 (R_a_/R_g_)	100	200 °C	[54]
Co_3_O_4_ crossed nanosheet (CNS)	5.6 (R_g_/R_a_)	100	111 °C	[55]
Pristine PEDOT:PSS	4.08% (ΔR/R_0_)	500	RT.	This work

## Methods

### SCC-DFTB Method and Models of PEDOT:PSS

The SCC-DFTB method is based on a second-order expansion of the DFT energy with respect to density fluctuations around a reference density [30]. The SCC-DFTB utilizes the Kohn-Sham orbitals with the optimized linear combination of atomic orbitals (LCAO) Slater-type valence electron basis set. The total energy of SCC-DFTB can be written as

1 $$ {E}_{SCC- DFTB}={\displaystyle \sum_{i\mu \nu}{c}_{\mu}^i}{c}_v^i{H}_{\mu v}^0+{\displaystyle \sum_{A> B}{E}_{A B}^{rep}}+\frac{1}{2}{\displaystyle \sum_{A B}{\gamma}_{A B}\varDelta {q}_A}\varDelta {q}_B $$

Where *μ* and *ν* denote atomic orbitals, A and B denote atoms, ${c}_{\mu}^i$ are the expansion coefficients of molecular orbitals, ${H}_{\mu v}^0$ is unperturbed Hamiltonian, ${E}_{AB}^{rep}$ is the two-body repulsive energy term, *Δq*_*A*_ and *Δq*_*b*_ are the induced charge on each atom A and B, respectively, and *γ*_*AB*_ is a distance-dependent function describing charge interactions.

**Fig. 1:**
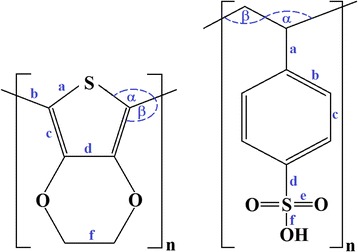
Molecular structures of **a** EDOT and **b** SS oligomers

Regarding SCC-DFTB, this method has been called as a “basis-set independent” method [31, 32]. There are no integrals calculated in the DFTB method, thus there cannot be a basis set superposition error (BSSE). In addition, different basis sets are usually derived for electronic and repulsive potential parameters, the effects of BSSE on PEDOT:PSS-NH_3_ interactions is therefore neglected for this study. The bond lengths, bond angle, and torsion angle of PEDOT and PSS are defined as shown in Fig. 1. To verify the accuracy of the SCC-DFTB method, the structure and electronic properties of PEDOT, PSS, and PEDOT:PSS (*n =* 1 to 3) obtained from SCC-DFTB method implemented on DFTB^+^ [33] in conjunction with the mio-0-1 parameter set [30, 34] were compared with density functional theory [35] at B3LYP/6-31G*[36, 37] level using GAMESS [38]. It should be noted that B3LYP can be well used for the description of the geometric and electronic structures of π-conjugated polymers [18, 19, 21]. However, it fails to accurately represent dispersion/weak non-covalent interactions. This leads to a serious limitation for investigation of PEDOT:PSS-NH_3_ interactions. The B3LYP was thus employed to study the geometric and electronic properties of PEDOT:PSS only. After validation of the SCC-DFTB accuracy, PEDOT, PSS, and PEDOT:PSS up to *n =*10 were fully optimized and studied based on SCC-DFTB calculation. Geometries were optimized until the atomic forces were less than 1.0 × 10^−4^ Hartree/Bohr. The SCC tolerance was set to 10^–6^ au. The electron temperature was kept to 1000 K in order to improve SCC convergence and include the effect of thermal electronic excitation [39, 40].

### QM/MD Simulation of EDOT:SS in Ammonia

**Fig. 2:**
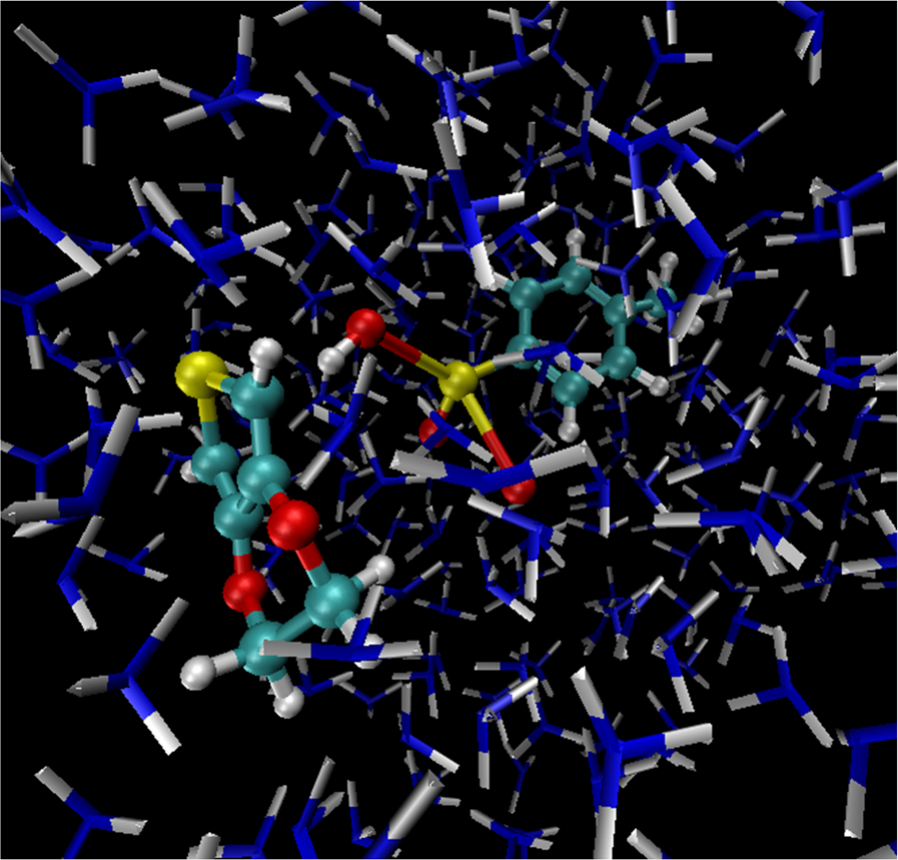
Simulation snapshot of EDOT:SS monomer in NH_3_ molecules at 298 K

The QM/MM simulation was performed under canonical ensemble. The system consists one EDOT:SS molecule and 250 NH_3_ molecules in a periodic cubic box of 16.38 × 16.38 × 16.38 nm^3^ as shown in Fig. 2. Total numbers of atoms in the simulation box were 1034 atoms. A target nuclear temperature of 298 K was maintained using a Berendsen thermostat [41]. The equations of motion were integrated using the Velocity Verlet algorithm [42] with an integration time step of 1 fs. The total simuation time were 100 ps.

### Fabrication of PEDOT:PSS Gas Sensor

The PEDOT:PSS aqueous solution (Clevios™ P VP AI 4083, solid content 1.3–1.7%, PEDOT:PSS weight ratio = 1:6) was purchased from Heraeus Precious Metals GmbH & Co., KG and used without any further purification. A PEDOT:PSS NH_3_ gas sensor was fabricated based on ink-jet printing method [17]. Briefly, interdigitated electrodes with 1-mm interdigit spacing were deposited on PET flexible substrate by screen printing of silver conductive paste. The aqueous PEDOT:PSS was mixed with dimethyl sulfoxide (DMSO), glycol (EG) and triton x-100 in order to improve conductivity, viscosity and surface tension. The mixed PEDOT:PSS electronic ink was then deposited on interdigitated electrodes by a modified ink-jet printer. The thickness of PEDOT:PSS sensing film could be controlled by varying the number of printed layers. The fabricated PEDOT:PSS gas sensor was tested with ammonia, acetone, ethanol, methanol, and toluene at 500 ppm concentration to assess the response and selectivity of the sensor. All experiments were performed at room temperature (25 ± 2 °C) and the relative humidity of 58 ± 2%. Gas response of PEDOT:PSS gas sensor is defined as

2 $$ S\left(\%\right)=\left(\frac{R_{\mathrm{gas}}-{R}_{\mathrm{air}}}{R_{\mathrm{air}}}\times 100\right), $$

where *R*_air_ and *R*_gas_ are the sensor resistance in pure air and in test gas, respectively.

## Results and Discussion

### Structural and Electronic Properties of PEDOT:PSS

**Fig. 3:**
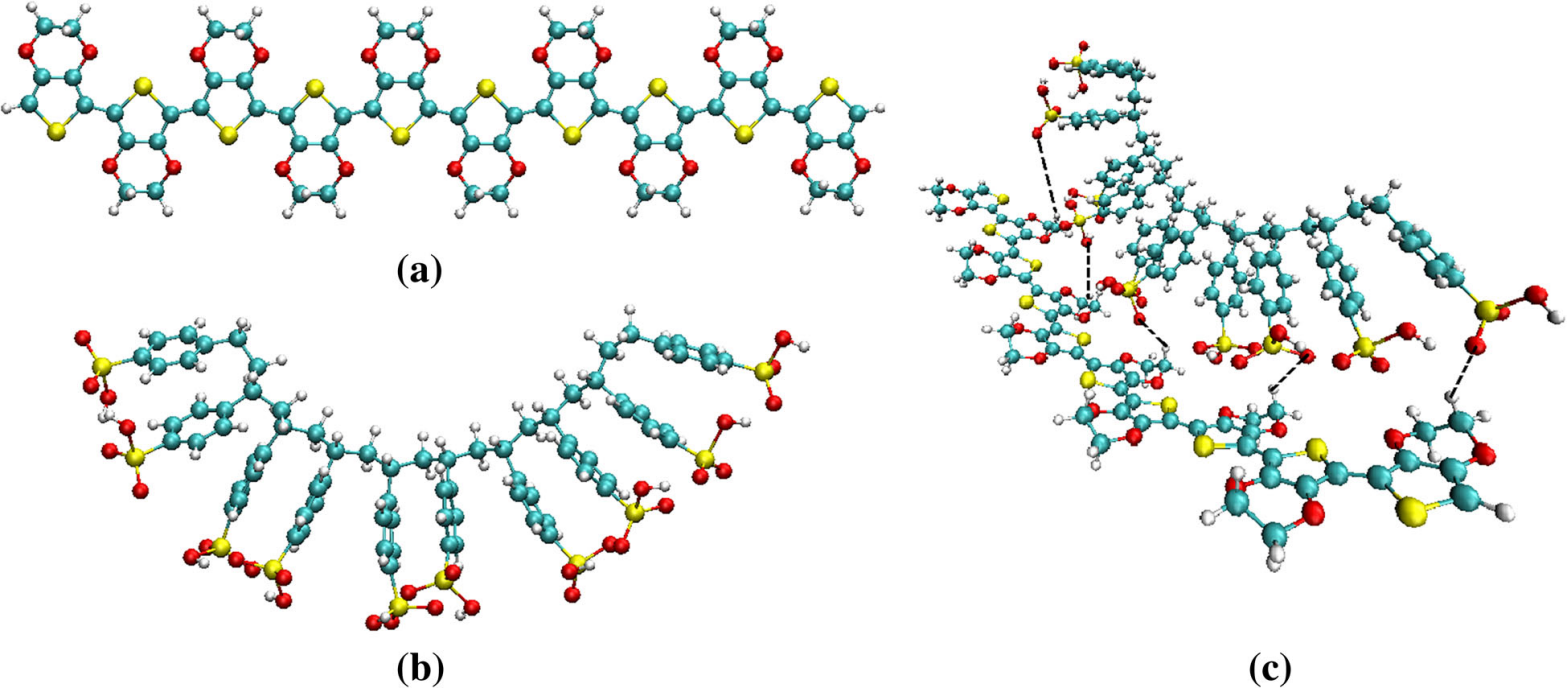
Optimized structures of **a** EDOT, **b** SS, and **c** EDOT:SS oligomers with *n =* 10 units based on SCC-DFTB calculation

List of bond lengths, bond angle, and torsion angle of EDOT, SS and EDOT:SS oligomers (*n =* 1–3) obtained at the SCC-DFTB and DFT methods is given in Additional file 1: Table S1–S3 in the supplementary data section. Root-mean-square deviations (RMSD) of bond lengths, bond angle and torsion angle of optimized structures (*n =* 1 to 3 units) between SCC-DFTB and B3LYP/6-31G* methods are shown in Table 2. The RMSD values were calculated by a simple equation; $RMSD=\sqrt{\frac{{\displaystyle \sum }{\left({X}_{DFTB}-{X}_{B3 LYP}\right)}^2}{n}}$, where X_DFTB_ and X_B3LYP_ are structural properties obtained by SCC-DFTB and B3LYP/6-31G* methods, respectively. It appears that these differences are quite small. The SCC-DFTB geometry is in good agreement with DFT method while calculation time of SCC-DFTB is ~1000 times faster than conventional DFT. To study the geometry of EDOT, SS, and EDOT:SS with increasing oligomers, it is found that average bond lengths of thiophene, quinonoid and benzenoid rings do not change significantly up to 10 oligomers (see Additional file 1: Table S1-S4 in the Supplementary data section). The optimized structures of EDOT, SS and EDOT:SS with *n =* 10 are displayed in Fig. 3. In EDOT:SS oligomers, the sulfonate functional groups of SS oligomers tends to interact with the EDOT oligomers. The H atoms of EDOT are closest to the O atoms of SS oligomers in all n units (*n =* 1–10). It indicates an important role of H-bonds formation (dash lines in Fig. 3c) in EDOT:SS oligomers. The average closest distance between EDOT and SS oligomers is found to be approximately 2.14 Å based on SCC-DFTB method. However, it should be noted that electrostatic interactions also dominate conformation of EDOT:SS oligomers. At 10-EDOT:SS oligomers, strong positive charges occurred at sulfurs atoms of SS oligomers are in range of 1.49e–1.56e while oxygen atoms of EDOT contribute average negative charges of 0.28 |e|. The existence of repulsive interactions between the sulfur atoms and attractive interactions between EDOT and SS oligomers cause a non-planar conformation in PEDOT:PSS chain structure. With increasing chain length, PEDOT:PSS exhibits coil-like conformation corresponding to the study by Gangopadhyay et al. [21] based on DFT calculation and experimental investigation by Kim et al. [43]

**Table 2:** Root mean square deviations (RMSD) of bond lengths, bond angle and torsion angle of optimized EDOT, SS and EDOT:SS structures (*n =* 1 to 3 units) between SCC-DFTB and B3LYP/6-31G* methods

	*n =* 1	*n =* 2	*n =* 3
Bond length (Å)	0.084	0.077	0.075
Bond angle (°)	1.128	1.960	0.621
Torsion angle (°)	-	2.218	0.771

**Fig. 4:**
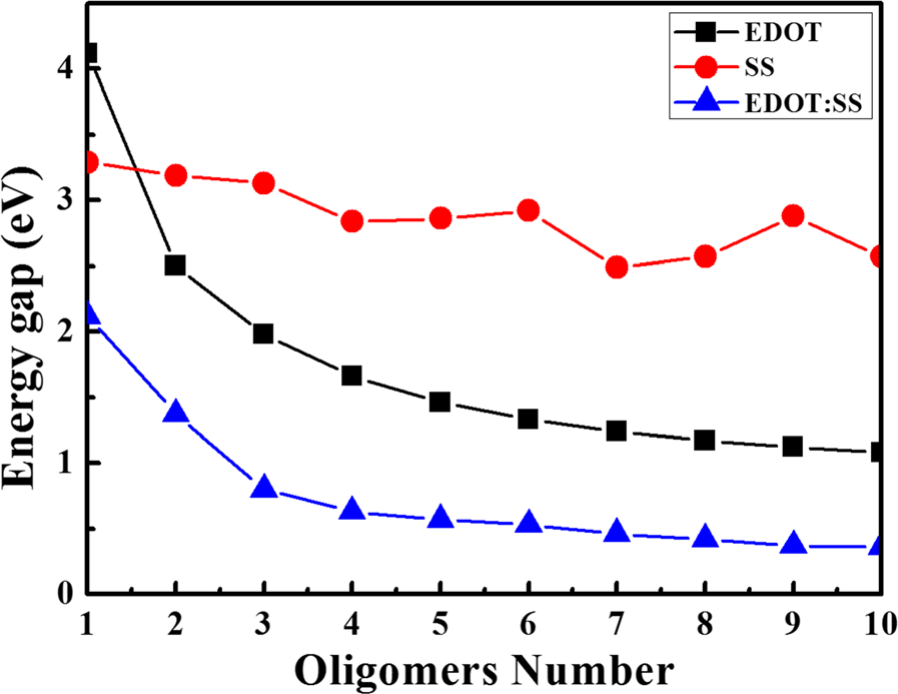
Variation of energy gaps of EDOT, SS, and EDOT:SS oligomers obtained by SCC-DFTB method

The HOMO, LUMO and energy gap (ε_g_) of EDOT, SS and EDOT:SS with *n =* 1–3 units based on B3LYP/6-31G* and SCC-DFTB methods are shown in Table 3. One can be seen that the ε_g_ of EDOT, SS and EDOT:SS (*n =* 1–3 units) predicted by the SCC-DFTB is less than that of B3LYP/6-31G* about 1.31–3.49 eV. Although there is a big difference ε_g_ prediction, the SCC-DFTB still yields values directly comparable with experimental results. For EDOT with eight units, B3LYP/6-31G* estimated the ε_g_ of 2.75 eV [20] while SCC-DFTB predicts the ε_g_ of 1.17 eV (see Fig. 4) which is in good agreement with experimental investigations (1.5–1.7 eV) [2, 44–46]. The HOMO and LUMO energies for EDOT, SS, and EDOT:SS with *n =* 1–10 units based on SCC-DFTB method are reported in Additional file 1: Table S5 in the supplementary data section.

**Table 3:** HOMO, LUMO and energy gap (ε_g_) in eV of EDOT, SS and EDOT:SS with *n =* 1–3 units obtained by B3LYP/6-31G* and SCC-DFTB methods

Model	n	B3LYP/6-31G*			SCC-DFTB		
		HOMO	LUMO	ε_g_	HOMO	LUMO	ε_g_
EDOT	1	−5.71	1.90	7.61	-5.38	-1.26	4.12
	2	−4.77	−0.71	4.06	−4.55	−1.96	2.59
	3	−4.33	−1.03	3.30	−4.2	−2.21	1.99
SS	1	−7.22	−1.09	6.13	−6.41	−3.11	3.30
	2	−7.20	−1.36	5.84	−6.53	−3.34	3.19
	3	−7.29	−1.48	5.81	−6.62	−3.49	3.13
EDOT:SS	1	−6.02	−0.89	5.13	−4.95	−2.83	2.12
	2	−4.67	−1.28	3.39	−4.16	−2.79	1.37
	3	−4.87	−1.58	3.29	−3.83	−3.04	0.79

The HOMO and LUMO energies can imply to the ionization potential and electron affinities, respectively [47]. For EDOT oligomers, the HOMO and LUMO energies increase and decrease, respectively, with increasing oligomers (n). These cause from an increase of *π* conjugation resulting to increase of electrical conductivity when number of oligomers increase (see Fig. 4). In case of SS oligomers, HOMO and LUMO energies do not increase/decrease linearly. These may come from variety of sulfonate functional groups conformation of SS oligomers. For EDOT:SS oligomers, it clearly shows enhancement of electrical conductivity in all n as shown in Fig. 4. At *n =* 10, the ε_g_ of EDOT:SS is 0.35 eV which is three times greater than that of pristine EDOT (1.08 eV). The electrons prefer to transfer from EDOT to SS oligomers ranging from 0.007 to 0.444 |e| with increasing oligomers (n).

### Sensing Property of PEDOT:PSS Gas Sensor

**Fig. 5:**
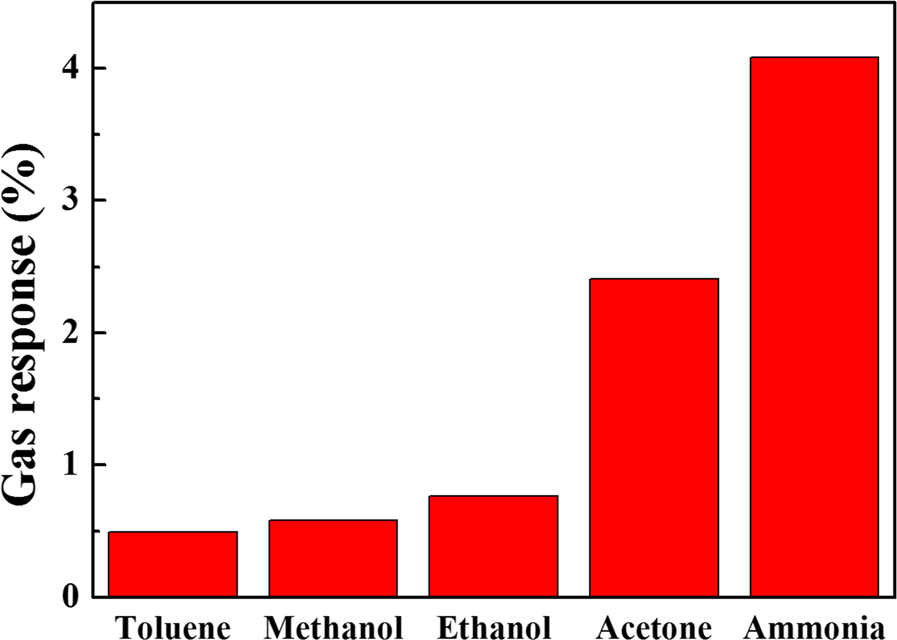
Gas response of the pristine PEDOT:PSS gas sensor to 500 ppm concentration of various VOCs at room temperature

The gas response of pristine PEDOT:PSS gas sensor to various volatile organic compound (VOCs) such as toluene, methanol, ethanol, acetone, and ammonia at room temperature is displayed in Fig. 5. It clearly shows that the pristine PEDOT:PSS gas sensor exhibited relatively high response and selectivity to ammonia compared with other VOCs. The gas responses to NH_3,_ acetone, methanol, ethanol, and toluene were 4.08, 2.41, 0.77, 0.58, and 0.49%, respectively. Sensing mechanism of PEDOT:PSS sensor to ammonia can be explained via direct charge transfer process and swelling process [17]. In this work, only direct charge transfer process has been investigated in depth based on SCC-DFTB method. The results will be discussed in the next section.

### QM/MD Simulation

**Fig. 6:**
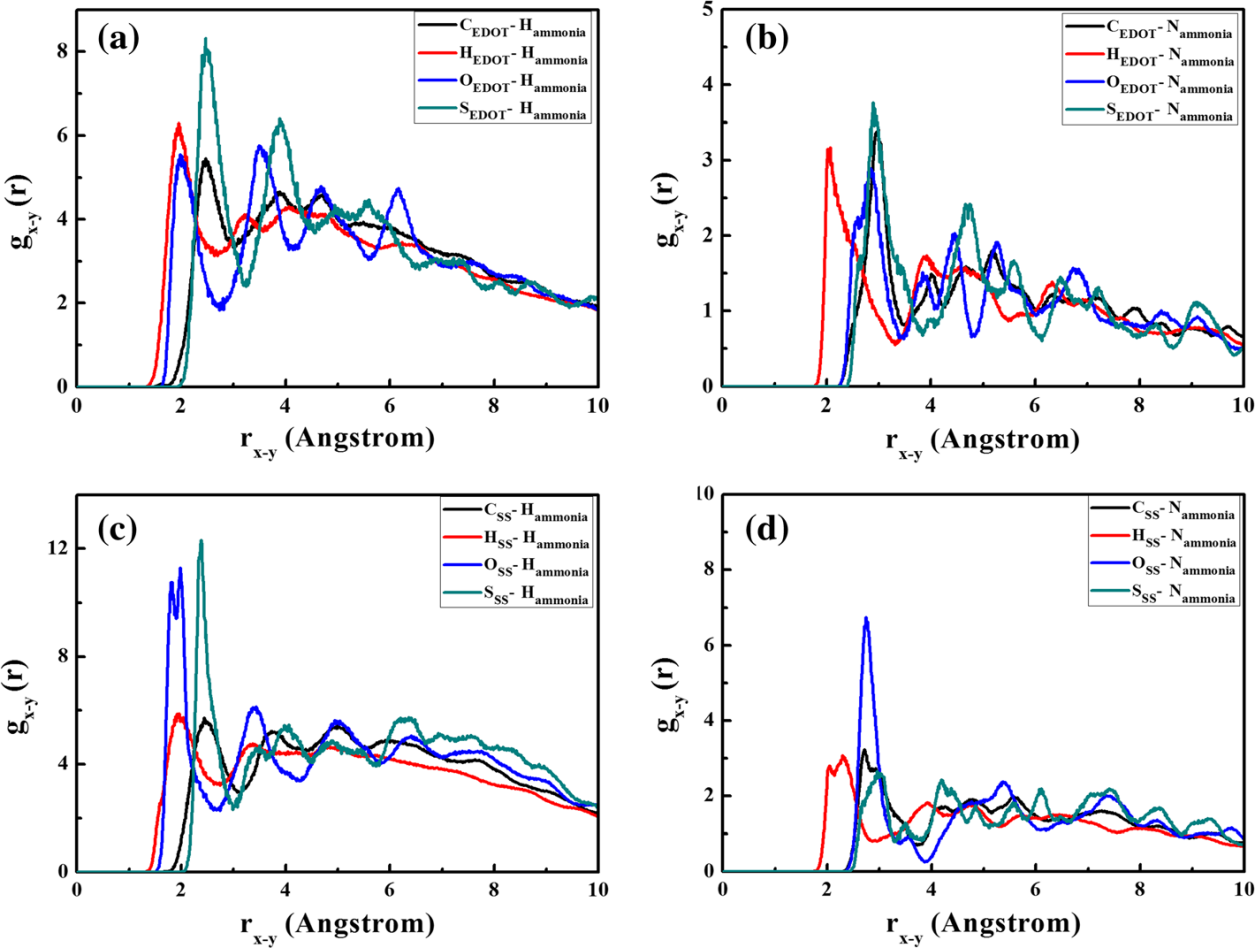
RDFs (g_x-y_(r)) between atoms of EDOT to **a** H atoms, **b** N atoms of NH_3_, atoms of SS to **c** H atoms, and **d** N atoms of NH_3_ molecules

**Fig. 7:**
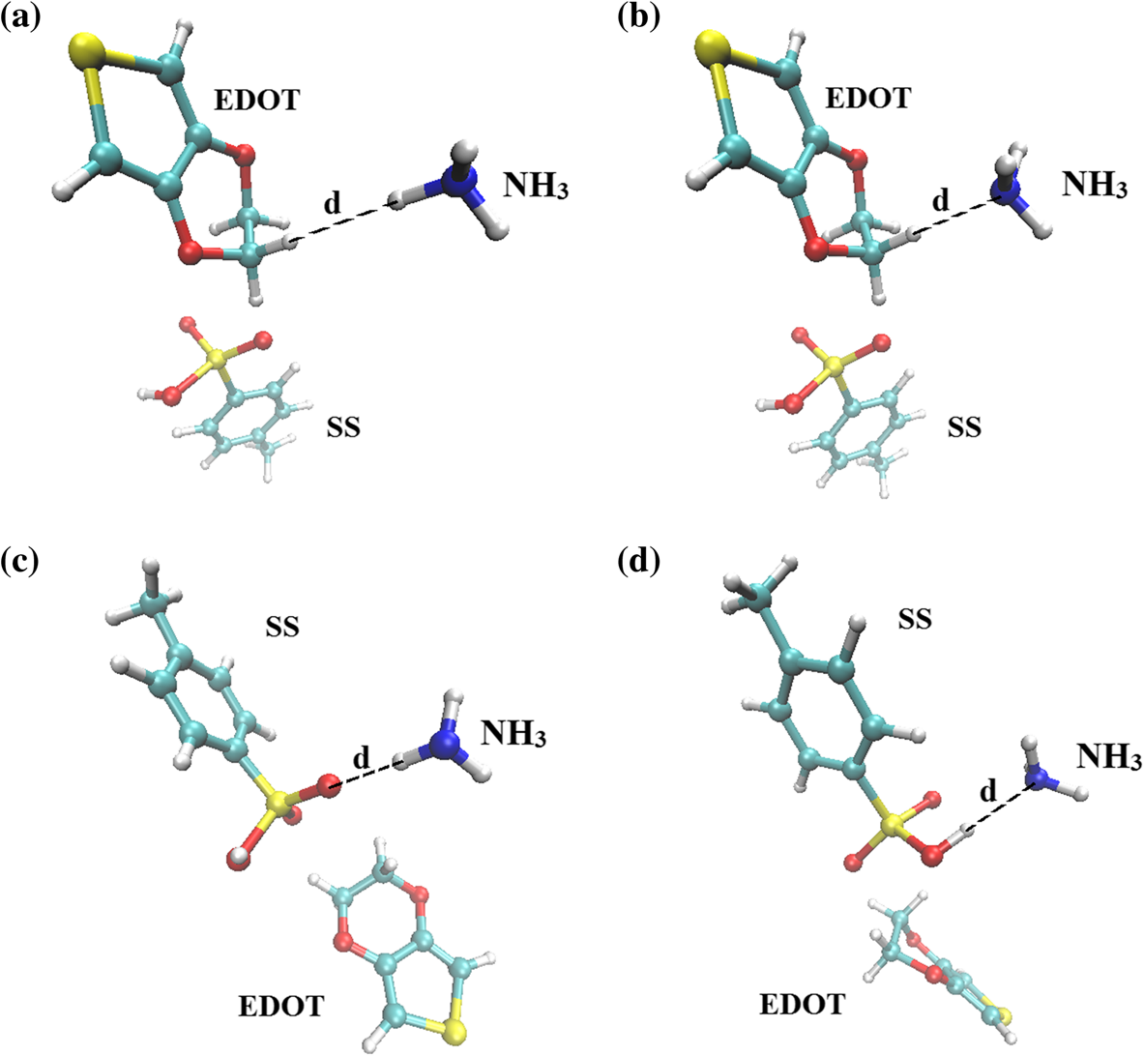
Orientations of NH_3_ molecules around EDOT:SS based on the first RDFs peaks

In order to study the tendency and behavior of NH_3_ orientation toward PEDOT:PSS, the QM/MD simulation of a EDOT:SS in 250 NH_3_ molecules was performed in a periodic box at room temperature. Last 50 ps simulation times were used for radial distribution function (RDF) analysis. The RDFs from the atoms of EDOT to the H and N atoms of NH_3_ molecules are shown in Fig. 6a and b, respectively. One can be seen that NH_3_ molecules prefer to localize at H atoms of EDOT molecule with the first RDFs peaks of 1.94 and 2.04 Å for H and N atoms of NH_3_ molecules, respectively. In case of SS, the probability of finding NH_3_ molecules surrounding the O atoms of SS is higher than that of the other atoms as displayed in Fig. 6c and d. Based on the first RDFs peaks, the H atoms of NH_3_ molecules turn toward the O atoms of SS at the position of 1.91 Å and the N atoms of NH_3_ tends toward the H atoms of SS at the position of 2.30 Å. The results suggest that NH_3_ molecules interact with both EDOT and SS and favor to bind at the sites of O and H atoms. To better understand the binding distances and interaction energies between EDOT:SS and NH_3_, four configurations (see Fig. 7) extracted from the first RDFs peaks were re-calculated with SCC-DFTB energy calculation including van der Waals dispersion corrections [48, 49]. The interaction energy (E_int_) can be calculated by the following equation:

3 $$ {E}_{\mathrm{int}}={E}_{tot}\left(\mathrm{EDOT}:\mathrm{SS}+{\mathrm{NH}}_3\ \right)-{E}_{tot}\left(\mathrm{EDOT}:\mathrm{SS}\right)-{E}_{tot}\left({\mathrm{NH}}_3\right), $$

where E_tot_(EDOT:SS+ NH_3_), E_tot_ (EDOT:SS) and E_tot_ (NH_3_) are the total energies of the EDOT:SS with NH_3_, individual EDOT:SS and individual NH_3_, respectively.

**Fig. 8:**
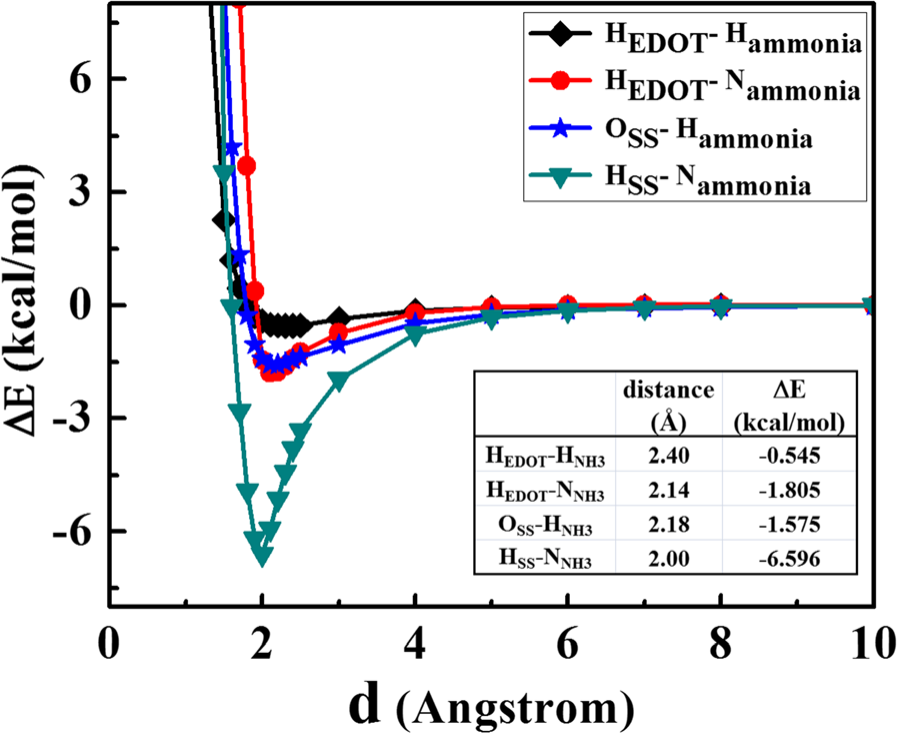
EDOT:SS-NH_3_ interaction energies at different adsorption sites and configurations as a function of the distance (*d*)

The interaction energy between EDOT:SS and NH_3_ at different adsorption sites and NH_3_ orientation configurations is shown in Fig. 8. The H_SS_-N_NH3_ configuration exhibits the highest interaction energy (6.596 kcal/mol) with the binding distance of 2.00 Å. This result suggests that the NH_3_ molecules prefers to interact with EDOT:SS via the lone pair on the N atom at H atoms of EDOT:SS. At this adsorption site, electron charge transfer was found to be from the NH_3_ molecule to the EDOT:SS (0.032 e). The holes of EDOT:SS interact with the electron-donating NH_3_. The delocalization degree of conjugated *π* electrons of EDOT:SS is increased by charge transfer from the adsorbed NH_3_ molecules. Formation of a neutral polymer backbone occurs and leads to decrease in charge carriers of EDOT:SS. It causes the increase in resistance of EDOT:SS in present of NH_3_. This behavior is in good agreement with our experimental results as shown in Fig. 5.

## Conclusions

The PEDOT:PSS conductive polymer for NH_3_ detection was investigated both experimentally and theoretically. The structural and electronic properties of PEDOT:PSS oligomers were studied based on SCC-DFTB method and compared with B3LYP/6-31 g*. Calculations indicated that SCC-DFTB is indeed capable of reproducing the DFT-predicted features of PEDOT:PSS conductive polymer system (C-S-O-H bonding). Non-planar conformation in PEDOT:PSS chain structure naturally occur due to the existence of repulsive interactions between the sulfur atoms and H-bond attractive interactions between EDOT and SS oligomers. The EDOT behaves as an electron donor for EDOT: SS composites. The electrical conductivity of EDOT increases with increasing oligomers and doping SS. The energy gap of EDOT: SS with 10 oligomers was found to be 0.35 eV based on SCC-DFTB. The printed PEDOT:PSS gas sensor exhibited good response and selective to NH_3_ at room temperature over VOCs such as toluene, methanol, ethanol, and acetone. Theoretical investigation showed interaction between NH_3_ and EDOT: SS via physisorption. The H atoms of SS are the most favorable adsorption site of NH_3_. Direct charge transfer process dominants changing in conductivity of EDOT:SS upon NH_3_ exposure at room temperature. The PEDOT:PSS sensor acts as an electron acceptor for NH_3_ detection. It is hoped that this work will be useful for better understanding of the NH_3_ interactions with PEDOT:PSS and can be used to confirm the direct charge transfer sensing mechanism of PEDOT:PSS gas sensors for NH_3_ detection.

## Additional Files

Additional file 1: Table S1. Average bond lengths, bond angle and torsion angle of EDOT, SS, EDOT of EDOT:SS (EDOT:SS^*1^) and SS of EDOT:SS (EDOT:SS^*2^) with *n* = 1 units optimized by B3LYP/6-31G* and SCC-DFTB calculation. **Table S2.** Average bond lengths, bond angle and torsion angle of EDOT, SS, EDOT of EDOT:SS (EDOT:SS^*1^) and SS of EDOT:SS (EDOT:SS^*2^) with *n* = 2 units optimized by B3LYP/6-31G* and SCC-DFTB calculation. **Table S3.** Average bond lengths, bond angle and torsion angle of EDOT, SS, EDOT of EDOT:SS (EDOT:SS^*1^) and SS of EDOT:SS (EDOT:SS^*2^) with *n* = 3 units optimized by B3LYP/6-31G* and SCC-DFTB calculation. **Table S4.** Average bond lengths, bond angle and torsion angle of EDOT, SS, EDOT of EDOT:SS (EDOT:SS^*1^) and SS of EDOT:SS (EDOT:SS^*2^) with *n* = 10 units optimized by SCC-DFTB calculation. **Table S5.** Energy of the HOMO and LUMO in eV of EDOT, SS and EDOT:SS oligomers optimized by SCC-DFTB calculation. (DOCX 29 kb)

## Abbreviations

EDOT: 3,4-ethylenedioxythiophene
NH_3_: Ammonia
PEDOT:PSS: Poly(3,4-ethylenedioxythiophene): poly(styrenesulfonate)
RDF: Radial distribution function
RMSD: Root mean square deviations
SCC-DFTB: Self-consistent charge density functional tight-binding
SS: Styrene sulfonate
