# Supplementary material for: Self-Consistent Charge Density Functional Tight-Binding Study of Poly(3,4-ethylenedioxythiophene): Poly(styrenesulfonate) Ammonia Gas Sensor
Source: Nanoscale Res Lett. 2017 Feb 6;12:90. doi: 10.1186/s11671-017-1878-2 (PMC5293716; doi:10.1186/s11671-017-1878-2)
Supplement: Additional file 1: Table S1. — Average bond lengths, bond angle and torsion angle of EDOT, SS, EDOT of EDOT:SS (EDOT:SS*1) and SS of EDOT:SS (EDOT:SS*2) with n = 1 units optimized by B3LYP/6-31G* and SCC-DFTB calculation. Table S2. Average bond lengths, bond angle and torsion angle of EDOT, SS, EDOT of EDOT:SS (EDOT:SS*1) and SS of EDOT:SS (EDOT:SS*2) with n = 2 units optimized by B3LYP/6-31G* and SCC-DFTB calculation. Table S3. Average bond lengths, bond angle and torsion angle of EDOT, SS, EDOT of EDOT:SS (EDOT:SS*1) and SS of EDOT:SS (EDOT:SS*2) with n = 3 units optimized by B3LYP/6-31G* and SCC-DFTB calculation. Table S4. Average bond lengths, bond angle and torsion angle of EDOT, SS, EDOT of EDOT:SS (EDOT:SS*1) and SS of EDOT:SS (EDOT:SS*2) with n = 10 units optimized by SCC-DFTB calculation. Table S5. Energy of the HOMO and LUMO in eV of EDOT, SS and EDOT:SS oligomers optimized by SCC-DFTB calculation. (DOCX 29 kb) [file 11671_2017_1878_MOESM1_ESM.docx]

**Supplementary Information for**

**Self-consistent charge density functional tight-binding study of poly(3,4-ethylenedioxythiophene):** **poly(styrenesulfonate) ammonia gas sensor**

Ampaiwan Marutaphan^1, 2^, Yotsarayuth Seekaew^1^ and Chatchawal Wongchoosuk^1,*^

^1^ Department of Physics, Faculty of Science, Kasetsart University, Chatuchak, Bangkok 10900,Thailand

^2^Faculty of Science and Technology, Rajamangala University of Technology Suvarnabhumi,

Nonthaburi 11000, Thailand

*Corresponding author >> E-mail: Chatchawal.w@ku.ac.th

Tel.: +662-562-5555; Fax: +662-942-8029

Table S1 Average bond lengths, bond angle and torsion angle of EDOT, SS, EDOT of EDOT:SS (EDOT:SS^*1^) and SS of EDOT:SS (EDOT:SS^*2^) with n=1 units optimized by B3LYP/6-31G* and SCC-DFTB calculation.

| Method | Model | a (Å) | b (Å) | c (Å) | d (Å) | e (Å) | f (Å) | α (°) | β (°) |
| --- | --- | --- | --- | --- | --- | --- | --- | --- | --- |
| B3LYP /6-31G* | EDOT | 1.74 | - | 1.36 | 1.44 | 1.37 | 1.52 | 121.34 | 127.76 |
|  | SS | 1.52 | 1.40 | 1.39 | 1.78 | 1.46 | 1.65 | 107.92 | 111.58 |
|  | EDOT:SS^*1^ | 1.74 | - | 1.37 | 1.44 | 1.36 | 1.52 | 121.23 | 127.53 |
|  | EDOT:SS^*2^ | 1.51 | 1.40 | 1.39 | 1.79 | 1.46 | 1.63 | 108.03 | 111.51 |
| SCC-DFTB | EDOT | 1.78 | - | 1.37 | 1.44 | 1.40 | 1.51 | 121.96 | 127.14 |
|  | SS | 1.50 | 1.40 | 1.39 | 1.82 | 1.65 | 2.00 | 106.71 | 109.67 |
|  | EDOT:SS^*1^ | 1.77 | - | 1.37 | 1.44 | 1.40 | 1.51 | 121.86 | 127.18 |
|  | EDOT:SS^*2^ | 1.48 | 1.40 | 1.39 | 1.84 | 1.64 | 1.88 | 109.92 | 109.84 |

Table S2 Average bond lengths, bond angle and torsion angle of EDOT, SS, EDOT of EDOT:SS (EDOT:SS^*1^) and SS of EDOT:SS (EDOT:SS^*2^) with n=2 units optimized by B3LYP/6-31G* and SCC-DFTB calculation.

| Method | Model | a (Å) | b (Å) | c (Å) | d (Å) | e (Å) | f (Å) | α (°) | β (°) | Torsion angle (°) |
| --- | --- | --- | --- | --- | --- | --- | --- | --- | --- | --- |
| B3LYP /6-31G* | EDOT | 1.76 | 1.44 | 1.38 | 1.43 | 1.37 | 1.52 | 122.59 | 128.27 | 179.88 |
|  | SS | 1.52 | 1.40 | 1.39 | 1.78 | 1.46 | 1.65 | 113.58 | 113.99 | 175.46 |
|  | EDOT:SS^*1^ | 1.77 | 1.44 | 1.38 | 1.43 | 1.37 | 1.52 | 122.53 | 128.31 | 175.22 |
|  | EDOT:SS^*2^ | 1.51 | 1.40 | 1.39 | 1.78 | 1.46 | 1.65 | 109.35 | 112.33 | 178.52 |
| SCC-DFTB | EDOT | 1.80 | 1.44 | 1.38 | 1.43 | 1.40 | 1.51 | 122.01 | 128.14 | 178.28 |
|  | SS | 1.50 | 1.40 | 1.40 | 1.84 | 1.64 | 1.95 | 109.22 | 114.22 | 179.42 |
|  | EDOT:SS^*1^ | 1.80 | 1.44 | 1.38 | 1.43 | 1.40 | 1.51 | 121.92 | 128.25 | 172.02 |
|  | EDOT:SS^*2^ | 1.50 | 1.40 | 1.40 | 1.85 | 1.64 | 1.88 | 108.75 | 117.81 | 178.63 |

Table S3 Average bond lengths, bond angle and torsion angle of EDOT, SS, EDOT of EDOT:SS (EDOT:SS^*1^) and SS of EDOT:SS (EDOT:SS^*2^) with n=3 units optimized by B3LYP/6-31G* and SCC-DFTB calculation.

| Method | Model | a (Å) | b (Å) | c (Å) | d (Å) | e (Å) | f (Å) | α (°) | β (°) | Torsion angle (°) |
| --- | --- | --- | --- | --- | --- | --- | --- | --- | --- | --- |
| B3LYP /6-31G* | EDOT | 1.75 | 1.44 | 1.38 | 1.43 | 1.37 | 1.52 | 122.01 | 128.06 | 179.98 |
|  | SS | 1.51 | 1.40 | 1.39 | 1.78 | 1.46 | 1.65 | 110.47 | 114.37 | 177.98 |
|  | EDOT:SS^*1^ | 1.76 | 1.44 | 1.37 | 1.43 | 1.37 | 1.52 | 121.92 | 128.15 | 177.87 |
|  | EDOT:SS^*2^ | 1.51 | 1.40 | 1.39 | 1.79 | 1.46 | 1.64 | 110.43 | 115.28 | 163.72 |
| SCC-DFTB | EDOT | 1.80 | 1.43 | 1.39 | 1.42 | 1.40 | 1.51 | 122.07 | 128.07 | 178.39 |
|  | SS | 1.51 | 1.40 | 1.40 | 1.84 | 1.68 | 1.87 | 109.22 | 114.22 | 177.73 |
|  | EDOT:SS^*1^ | 1.80 | 1.43 | 1.39 | 1.42 | 1.40 | 1.51 | 121.96 | 128.17 | 177.86 |
|  | EDOT:SS^*2^ | 1.51 | 1.40 | 1.40 | 1.84 | 1.64 | 1.88 | 111.81 | 113.63 | 155.56 |

Table S4 Average bond lengths, bond angle and torsion angle of EDOT, SS, EDOT of EDOT:SS (EDOT:SS^*1^) and SS of EDOT:SS (EDOT:SS^*2^) with n=10 units optimized by SCC-DFTB calculation.

| Method | Model | a (Å) | b (Å) | c (Å) | d (Å) | e (Å) | f (Å) | α (°) | β (°) | Torsion angle (°) |
| --- | --- | --- | --- | --- | --- | --- | --- | --- | --- | --- |
| SCC-DFTB | EDOT | 1.80 | 1.43 | 1.39 | 1.42 | 1.40 | 1.51 | 122.10 | 128.1 | 177.9 |
|  | SS | 1.51 | 1.40 | 1.40 | 1.48 | 1.66 | 1.89 | 109.26 | 114.14 | 168.36 |
|  | EDOT:SS^*1^ | 1.80 | 1.43 | 1.39 | 1.42 | 1.40 | 1.51 | 122.16 | 128.04 | 176.66 |
|  | EDOT:SS^*2^ | 1.51 | 1.40 | 1.40 | 1.84 | 1.64 | 1.88 | 109.73 | 115.29 | 145.15 |

Table S5 Energy of the HOMO and LUMO in eV of EDOT, SS and EDOT:SS oligomers optimized by SCC-DFTB calculation.

| Oligomers Number (n) | EDOT | | SS | | EDOT:SS | |
| --- | --- | --- | --- | --- | --- | --- |
|  | HOMO | LUMO | HOMO | LUMO | HOMO | LUMO |
| 1 | -5.38 | -1.26 | -6.41 | -3.11 | -4.95 | -2.83 |
| 2 | -4.55 | -1.96 | -6.53 | -3.34 | -4.16 | -2.79 |
| 3 | -4.2 | -2.21 | -6.62 | -3.49 | -3.83 | -3.04 |
| 4 | -4.00 | -2.34 | -6.44 | -3.6 | -3.71 | -3.08 |
| 5 | -3.88 | -2.41 | -6.51 | -3.66 | -3.6 | -3.04 |
| 6 | -3.79 | -2.46 | -6.55 | -3.63 | -3.59 | -3.06 |
| 7 | -3.73 | -2.49 | -6.49 | -4.00 | -3.58 | -3.12 |
| 8 | -3.68 | -2.51 | -6.59 | -4.02 | -3.43 | -3.01 |
| 9 | -3.65 | -2.53 | -6.58 | -3.69 | -3.48 | -3.11 |
| 10 | -3.62 | -2.54 | -6.63 | -4.06 | -3.49 | -3.14 |
